# Metabolic Engineering of the Isopentenol Utilization Pathway Enhanced the Production of Terpenoids in *Chlamydomonas reinhardtii*

**DOI:** 10.3390/md20090577

**Published:** 2022-09-15

**Authors:** Mei-Li Zhao, Wen-Sheng Cai, Si-Qi Zheng, Jia-Lin Zhao, Jun-Liang Zhang, Ying Huang, Zhang-Li Hu, Bin Jia

**Affiliations:** 1 Guangdong Technology Research Center for Marine Algal Bioengineering, Guangdong Provincial Key Laboratory for Plant Epigenetics, Shenzhen Engineering Laboratory for Marine Algal Biotechnology, Longhua Innovation Institute for Biotechnology, College of Life Sciences and Oceanography, Shenzhen University, Shenzhen 518060, China; 2 College of Physics and Optoelectronic Engineering, Shenzhen University, Shenzhen 518060, China

**Keywords:** microalgae, *Chlamydomonas reinhardtii*, terpenoid, isopentenol utilization pathway, limonene

## Abstract

Eukaryotic green microalgae show considerable promise for the sustainable light-driven biosynthesis of high-value fine chemicals, especially terpenoids because of their fast and inexpensive phototrophic growth. Here, the novel isopentenol utilization pathway (IUP) was introduced into *Chlamydomonas reinhardtii* to enhance the hemiterpene (isopentenyl pyrophosphate, IPP) titers. Then, diphosphate isomerase (IDI) and limonene synthase (MsLS) were further inserted for limonene production. Transgenic algae showed 8.6-fold increase in IPP compared with the wild type, and 23-fold increase in limonene production compared with a single *MsLS* expressing strain. Following the culture optimization, the highest limonene production reached 117 µg/L, when the strain was cultured in a opt2 medium supplemented with 10 mM isoprenol under a light: dark regimen. This demonstrates that transgenic algae expressing the IUP represent an ideal chassis for the high-value terpenoid production. The IUP will facilitate further the metabolic and enzyme engineering to enhance the terpenoid titers by significantly reducing the number of enzyme steps required for an optimal biosynthesis.

## 1. Introduction

Terpenoids have a wide range of applications as pharmaceuticals due to their anticancer (paclitaxel), antimalarial (artemisinin), antiviral (betulinic acid) and antibiotic (fumigaclavine A) properties [1]. They are also a versatile source of commercial compounds, including fragrances (limonene), biofuels (bisabolane), pigments (carotenoids), flavorings (guaiol), biopolymers (rubber) and pesticides (pyrethrin I) [2]. All naturally produced terpenoids are synthesized from just two five-carbon (C5) hemiterpene diphosphates, isopentenyl diphosphate (IPP) and its highly electrophilic isomer, dimethylallyl diphosphate (DMAPP), by a sequential head-to-tail condensation [3].

Two natural pathways for the biosynthesis of IPP and DMAPP are the cytosolic mevalonate (MVA) and chloroplastic 2-C-methyl-D-erythritol 4-phosphate (MEP) (also known as 1-deoxy-D-xylulose 5-phosphate (DOX)) pathways [2,4]. The MVA pathway, which is utilized in archaea, eukaryotes, and some bacteria, consists of six enzymatic steps that start with acetyl-CoA in the cytosol. IPP, synthesized via the MVA pathway, is isomerized by isopentenyl diphosphate isomerase (IDI) to form DMAPP [4,5]. Within most bacteria, green algae and cyanobacteria, the MEP pathway consists of seven enzymatic steps that start with the condensation of pyruvate and D-glyceraldehyde 3-phosphate (GAP) to produce IPP and DMAPP at a ratio of 5:1 in the plastid [4,5].

Eukaryotic green microalgae are especially promising as innovative green cell-factory biocatalysts for the sustainable light-driven production of heterologous high-value terpenoids. Their fast and inexpensive phototrophic growth combined with the availability of mature genetic engineering tools means they are increasingly being viewed as alternatives to established heterotrophic hosts [6,7]. *Chlamydomonas reinhardtii* is the prototypical microalgal model species since it is fast-growing and photoautotrophic, i.e., it uses sunlight and carbon dioxide to produce organic materials, and is readily cultivatable in inexpensive water-based media. It is thus a potentially excellent natural route for the efficient production of high-value terpenoids, such as the sesquiterpenes (E)-α-bisabolene and patchoulol, and the diterpenoid 13*R*(+) manoyl oxide [6,8]. Indeed, several recent reports have confirmed that *C. reinhardtii* is capable of heterologous terpenoid production [9,10,11].

*C. reinhardtii* relies solely on the MEP pathway in the chloroplast to supply C5 terpenoid precursors [4,9,10,11]. However, the weak carbon fluxes through the native MEP pathway limit the IPP and DMAPP titers, which may impede the production efficiency of high-value terpenoid products via this route [12]. To increase the isoprenoid production, the titers of IPP and DMAPP need to be enhanced by increasing the carbon flux towards the MEP pathway [13]. Numerous rational design studies have previously targeted bottleneck steps within the MEP pathway, but these have had little positive impact on target production [10] or have negatively affected product levels, e.g., in the case of amorpha-4,11-diene [14]. The MEP pathway is highly interconnected with other elements of the cellular metabolism, which may be the reason for the multiple bottlenecks that impose constraints on rational engineering [15]. Low transgene expression rates and difficulties in expressing multiple genes in algal strains are further limiting factors [10,11]. As a result, a simpler, more effective approach to the terpenoid precursor metabolic engineering to enhance the high-value terpenoid production is badly needed.

The recent development of novel alcohol-dependent hemiterpene (ADH) biosynthetic pathways to produce terpenoid precursors, which circumvent the native metabolism, offers an attractive opportunity [3]. These ADH pathways are much simpler than the native MVA and MEP pathways and comprise just two sequential phosphorylation steps. The first step produces DMAP or IP by phosphorylating dimethylallyl alcohol, prenol or isoprenol; the second step produces DMAPP and IPP by phosphorylating DMAP or IP [3,16,17,18]. The concise and robust nature of the ADH pathways significantly reduces the difficulties involved with the multiple gene engineering in *C. reinhardtii*, and decouples the DMAPP/IPP synthesis from the complex native metabolic regulation. This ensures that the carbon flux required for the terpenoid biosynthesis is not in competition with that required for cell growth [3,16,17,18].

**Figure 1:**
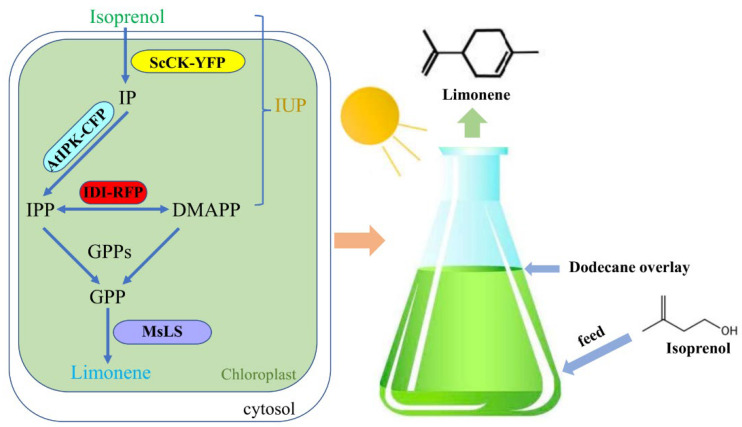
Metabolic pathway design for the biosynthesis of limonene from isopentenol isomers in engineered *Chlamydomonas reinhardtii*. The fluorescent protein fusions allowed direct screening for expressing strains by using a microplate reader. AtIPK: *Arabidopsis thaliana* isopentenyl phosphate kinase; CFP: mCerulean3 (cyan) fluorescent protein; DMAPP: dimethylallyl diphosphate; GPP: geranyl diphosphate; GPPs: GPP synthase; IDI: diphosphate isomerase; IP: isopentenyl monophosphate; IPP: isopentenyl pyrophosphate; IUP: isopentenol utilization pathway; MsLS: *Mentha spicata* limonene synthase; RFP: mRuby2 (red) fluorescent protein; ScCK: *Saccharomyces cerevisiae* choline kinase; YFP: mVenus (yellow) fluorescent protein.

In this study, we introduced a nonnative C5 terpenoid precursor production pathway, the isopentenol utilization pathway (IUP) (an ADH pathway), into *C. reinhardtii*. By introducing the IUP into the chloroplast of *C. reinhardtii*, the transgenic algae showed a clear increase in IPP/DMAPP titers, and thus can be used as an ideal chassis for the high-value terpenoid production. Additionally, we further characterized the utility of the pathway by the sequential transformation with the isopentenyl diphosphate isomerase (IDI) and limonene synthase (LS) genes into the chloroplast of *C. reinhardtii* to produce monoterpenoid limonene (Figure 1). This study first tried to confirm that the IUP could be used as a two-step shortcut for IPP synthesis in the *C. reinhardtii* chloroplast. This provides an effective approach to increase the production of limonene in *C. reinhardtii* and might also be suitable for the biological production of other high-value terpenoid. Moreover, the isoprenol precursor needs to cross cell walls, cell membranes and chloroplast membranes to be used by the IUP, this cannot be demonstrated in *E. coli* or cyanobacterium. Finally, we evaluated the culture parameters for the production of limonene from *C. reinhardtii*, including the capacity of a dodecane solvent overlay for the extraction of limonene. In engineered *C. reinhardtii*, hemiterpene diphosphate accumulation can be greatly improved by the IUP, and can robustly produce terpenoid-limonene by isoprenol feeding. What’s more, the IUP engineering will facilitate the production of high-value terpenoids in *C. reinhardtii*.

## 2. Results

### 2.1. Construction of the IUP in C. reinhardtii

**Figure 2:**
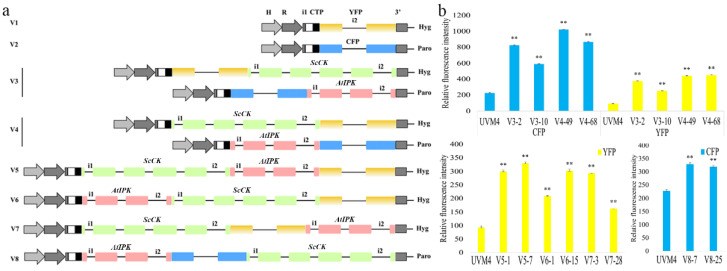
Overview of the expression and screening strategy for the C5 hemiterpene diphosphate production in *C. reinhardtii*. (**a**) Expression vectors for the fluorescent reporters YFP and CFP (V1 and V2, respectively); fusion of the codon-optimized choline kinase (CK) and isopentenyl phosphate kinase (IPK) to the C-terminus (V3) or N-terminus (V4) of mCerulean3 and the mVenus reporters in the pOpt_mCerulean3_Paro and pOpt_mVenus_Hyg expression vectors; fusion of CK and IPK with YFP or CFP in various arrangements (V5, 6, 7, and 8). All constructs in this work were transformed into *C. reinhardtii* strains and recovered under selective antibiotic conditions, as depicted. (**b**) Microplate reader-screened strains expressing fluorescent fusion proteins. Three biological replicates were used to calculate the fluorescence intensity averages and the standard deviations (SD) in all cases. Statistical significance (one-way ANOVA) compared to UVM4 is represented by asterisks (** indicates a difference at the *p* ≤ 0.01 level).

The codon-optimized ScCK and AtIPK genes were separately expressed or fused in a frame with YFP (mVenus) or CFP (mCerulean) to increase the efficiency of the identification of the transformants showing ScCK and/or AtIPK expressions (Figure 2a). Thus, the transformants with a high expression level of the key enzymes of the IUP could be screened according to the fluorescence level. ScCK and AtIPK were designed to contain a short and flexible GSGSGS or GSGS linker peptide at their C-termini to minimize any steric effects the fusion may have. It has been demonstrated that the fusion of consecutive enzymes in a metabolic pathway into a multi-activity protein can be beneficial for the effective substrate utilization and thereby increase product yields [9,19,20,21,22]. Therefore, we also tested whether the fusion of ScCK and AtIPK into a single polypeptide had a positive impact on the C5 hemiterpene diphosphate production (Figure 2a). As a result, eight versions of constructs were generated. 2twoconstructs (V1 and V2) only contained the fluorescent proteins. V3 represents ScCK and AtIPK that were separately fused at the C-terminal end of YFP (mVenus) or CFP (mCerulean) (YFP-ScCK and CFP-AtIPK). V4 represents ScCK and AtIPK that were separately fused at the N-terminal end of YFP (mVenus) or CFP (mCerulean) (ScCK-GSGSGS-YFP and AtIPK-GSGSGS-CFP). V5 represents the fusion enzyme ScCK-GSGSGS-AtIPK-GSGSGS-YFP. V6 represents the fusion enzyme AtIPK-GSGSGS-ScCK-GSGSGS-YFP. V7 represents the fusion enzyme ScCK-GSGSGS- YFP-AtIPK. V8 represents the fusion enzyme AtIPK-GSGSGS-CFP-ScCK.

For each gene expression construct, we randomly selected at least 300 transformants and used a microplate reader to assess the fluorescence signal of the transformants in the mid-logarithmic phase of the culture. The strains V3-2, V3-10, V4-49, V-68, V5-1, V5-7, V6-1, V6-15, V7-3, V7-28, V8-7 and V8-25, which had higher fluorescence intensities than other strains generated from the same vector (Figure 2b), were chosen for the quantitative analysis of IPP by HPLC-MS/MS.

### 2.2. IUP Enhanced the C5 Hemiterpene Diphosphate Production

**Figure 3:**
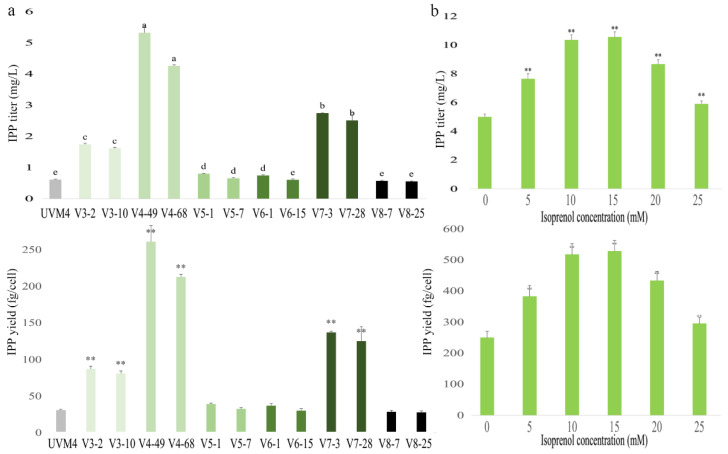
IPP production in transgenic algae. (**a**) IPP detected by LC-MS/MS in the extraction agent from cultivated algal cells. Three biological replicates were used to calculate the IPP yield averages and SDs. One-way ANOVA was used for the statistical analyses. Different letters (a–e) indicate significant differences (*p* ≤ 0.01). (**b**) IPP production of transgenic algae (V4-49) grown in a TAP medium supplemented with various concentrations of isoprenol. Three biological replicates were used to calculate the IPP yield averages and SDs. Statistical significance (one-way ANOVA) compared to 0 mM is represented by asterisks (** indicates a difference at the *p* ≤ 0.01 level).

Of all the vector constructs, the transformants which contained the V4 construct, showed relatively higher IPP productivities, with the strains V4-49 and V4-68 being the best (Figure 3a). Strain V4-49, which co-expressed both enzymes individually and at the N-terminal end of the fusion (i.e., ScCK-GSGSGS-YFP and AtIPK-GSGS-CFP), gave the greatest increase in production, achieving 5.3 mg/L IPP, which was 8.6-fold higher than that produced by the parental UVM4 strain (Figure 3a). In addition, we also noticed that the fusion order had a significant impact on the folding and function of the fusion enzymes. Thus, strain V4-49 gave a 3-fold higher IPP yield than that produced by the strain V3-2 in which the individual enzymes were at the C-terminal end of the fusion proteins, i.e., YFP-ScCK and CFP-AtIPK (Figure 3a). These results indicate that the individual expression of each enzyme and an N-terminal orientation in the fusion with YFP/CFP gives a superior product generation.

Among the V5, V6, V7 and V8 constructs, in which the ScCK and AtIPK are expressed together in a single fusion protein, the V7-3 and V7-28 strains gave a higher IPP yield. ScCK-GSGSGS-YFP-AtIPK (strain V7-3) exhibited the highest production, achieving 2.7 mg/L IPP, which was 4-fold higher than that of the parental UVM4 strain. Similar to the UVM4, the V8 (AtIPK-GSGS-CFP-ScCK) strains had a low fluorescence intensity compared with other strains, suggesting that V8 strains may have lower expression levels of ScCK and AtIPK, resulting in lower IPP productivities. The results indicate that individual expression of the two enzyme activities, albeit as fusion proteins with fluorescent proteins, is better than the incorporation of both activities in the same protein. Some previous studies also showed that the direct fusion of proteins can result in an impaired biological activity, probably because the functional domains are brought too close together to properly interact with their corresponding binding proteins (i.e., receptors or ligands) [23] or because of other steric effects.

To determine the toxicity of the reaction substrates in *C. reinhardtii*, UVM4 cells were cultured in a TAP medium supplemented with different concentrations of isoprenol or prenol. *C. reinhardtii* can survive, but not grow, in a TAP medium supplemented with a maximum of 30 mM isoprenol or 20 mM prenol, while a concentration of 20 mM isoprenol or 10 mM prenol is suitable for the growth of *C. reinhardtii* (Supplementary Figure S1). On the one hand, the concentration of isoprenol affects the growth of *C. reinhardtii*; on the other hand, it will affect IPP production. Therefore, it is necessary to explore the optimal substrate concentration. The transformant was cultured in a TAP medium supplemented with different concentrations of isoprenol until the mid-logarithmic phase or stationary phase was reached, and the IPP content was quantified. The highest IPP content was produced by the transformant V4-49 that was cultured in the TAP medium supplemented with 15 mM isoprenol (Figure 3b). These results indicate that the substrate (isoprenol or prenol) can enhance the IPP production at certain concentrations without influencing the development of *C. reinhardtii* strains heterologously expressing the IUP.

### 2.3. Heterologous Expression of LS in the IUP Strain Improves Limonene Production

We further demonstrated the utility of the IUP by combining it with a downstream module for the limonene synthesis. Limonene, a cyclic monoterpene, possesses citrus-like olfactory properties and multiple physiological functions [24]. Limonene synthase (LS) was chosen from spearmint (*Mentha spicata*) because of its high fidelity, i.e., >90% of the LS product is limonene with minimal levels of isomeric by-products [25]. To allow catalytic activity in the cytosol, the predicted chloroplast transit peptide (CTP) of LS was replaced by methionine for the translation initiation, while retaining residues R58/R59 [5,26]. The precursor of limonene is geranyl diphosphate (GPP), which is condensed from one molecule of dimethylallyl pyrophosphate (DMAPP) and one molecule of isopentenyl pyrophosphate (IPP). Thus, the IPP/DMAPP ratio plays an essential role in the regulation of limonene production. To balance the ratio of IPP and DMAPP, isopentenyl diphosphate isomerase (IDI) was chosen [16]: IDI is a key rate-limiting enzyme in terpenoid biosynthesis that performs the regulatory isomerization of IPP into DMAPP and vice versa [27].

**Figure 4:**
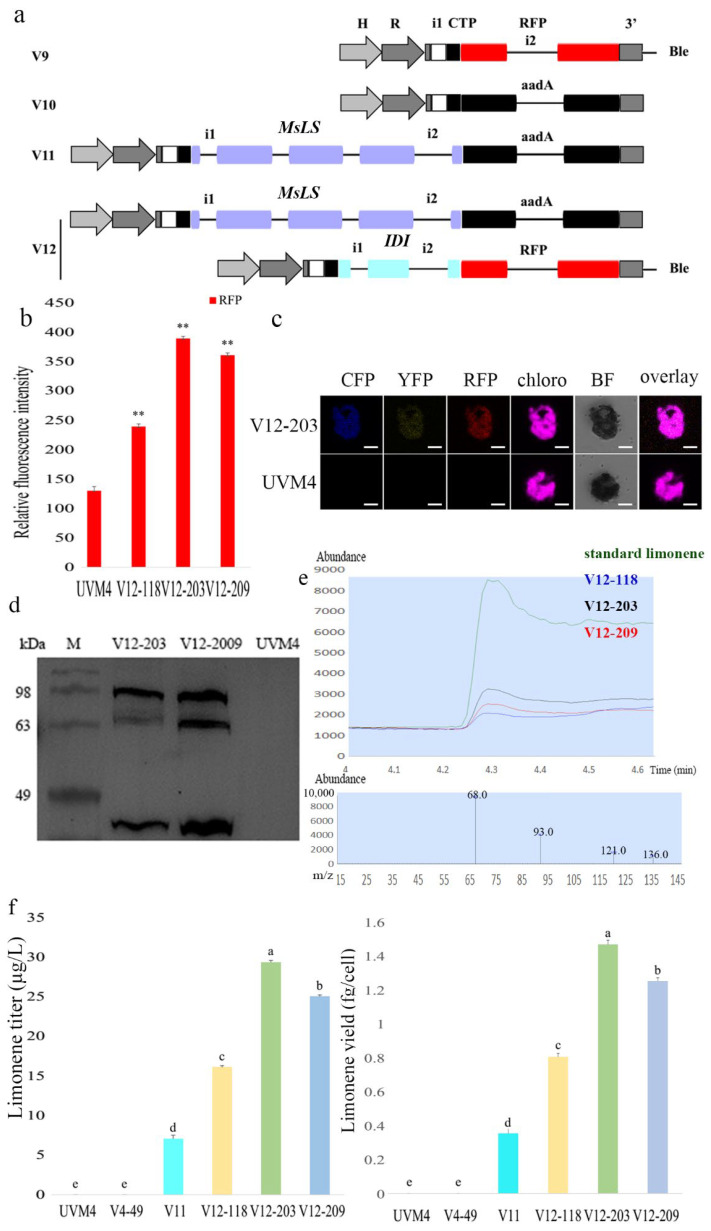
Overview of the expression and screening strategy for the limonene production in *C. reinhardtii*. (**a**) Expression vectors for the fluorescent reporter RFP (V9) and streptomycin 3″-adenylyltransferase (aadA) (V10); expression vector for the fusion of the codon-optimized limonene synthase (LS) to the N-terminus of aadA (V11); expression vectors for the fusion of LS and isopentenyl diphosphate isomerase (IDI) to the N-terminus of aadA and mRuby2 in the pOpt_aadA and pOpt_mRuby2_Ble constructs, respectively (V12). All constructs in this work were transformed into *C. reinhardtii* strains and recovered under selective antibiotic conditions, as depicted. (**b**) Microplate reader-screened strains expressing fluorescent fusion proteins. Three biological replicates were used to calculate the fluorescence intensity averages and SDs. Statistical significance (one-way ANOVA) compared to UVM4 is represented by asterisks (** indicates a difference at the *p* ≤ 0.01 level). (**c**) Fluorescence microscopy images of the parental strain (UVM4) and representative individual cells co-expressing ScCK-YFP, AtIPK-CFP, IDI-RFP and MsLS-aadA. Scale bars represent 5 µm. The PsaD chloroplast target peptide (CTP) was used for all expression constructs. The YFP, CFP, and RFP, could be targeted effectively to the algal chloroplast with this CTP. (**d**) Western blot of a total cellular protein with an α-StrepII tag antibody. V12-203 and V12-209 expressing strains exhibit signals at the appropriate predicted molecular mass (~ 92/88, 62, and 47 kDa). The predicted molecular masses of ScCK-YFP (~92 kDa) and MsLS-aadA (~88 kDa) were basically the same. So, three bands were detected. M—marker. (**e**) GC elution profiles and mass spectra of limonene and dodecane samples. (**f**) Limonene was detected by GC-MS in dodecane samples used as the organic-phase overlay in shake flasks. No peak for limonene was detectable in dodecane samples from the parental strain (UVM4 and V4-49) and a dodecane control. Three biological replicates were used to calculate limonene yield averages and SDs. One-way ANOVA was used for the statistical analyses. Different letters (a–e) indicate significant differences (*p* ≤ 0.05).

The V4-49 strain, a high-level C5 hemiterpene diphosphate-producing background strain, was chosen for the limonene synthesis. As mentioned above, the co-expression of the individual enzymes (ScCK-GSGSGS-YFP and AtIPK-GSGS-CFP) and the N-terminal orientation in the fusion with YFP/CFP was determined to most favor product generation. Thus, we chose a new vector, pOpt2_PsaD_aadA_Min, which confers spectinomycin resistance on *C. reinhardtii*, for the selection of transformants, and allowed the construction of N-terminal orientations of the enzyme IDI in fusion with RFP [11] and MsLS in fusion with aadA (Figure 4a). We randomly selected at least 300 transformants of each gene expression construct, using a microplate reader to detect the fluorescence signal of the transformants in the mid-logarithmic phase. The V12-118, V12-203 and V12-209 strains showed higher fluorescence intensities than other strains (Figure 4b), and the V12-203 strains showed the highest fluorescence intensity. The fluorescence signals of V12-203 were visualized by confocal microscopy. The fluorescence signals of all the fusion proteins were completely overlapped with those of the chlorophyll (Figure 4c), which indicated that the fusion proteins were localized to the chloroplast as intended by the CTP. Furthermore, the fusion proteins exhibited appropriate molecular masses in western blotting (Figure 4d).

In order to promote terpene production and accelerate its detection, dodecane was overlayed to the strain culture because of its relatively low volatility, compatibility with algal growth and ability to solvate terpenoids [8,26]. The dodecane phase was harvested and subjected to GC-MS analysis (Figure 4e,f). The standard calibration curves in the range of 0.5–5 ppm limonene in dodecane were used to quantify the amount of limonene, and the R^2^ coefficient for the calibration was greater than 0.99 (standard curve in Supplementary Figure S7). The quantification of limonene was performed using a standard curve of the commercial (R)-limonene (Merck KGaA, Darmstadt, Germany). The strains, and the transformants which contained the V12 construct, gave the highest limonene production, achieving approximately 23 µg/L limonene from the strain V12 fermentations, which was approximately 23-fold that of V11 (which is cultured in the TAP medium without isoprenol added, MsLS expressed alone, as a control). The results show that the expression of the artificial IUP in *C. reinhardtii* carrying the MsLS gene leads to an enhancement of the limonene production by more than 23-fold, likely due to the increased carbon flux towards IPP and DMAPP. The marked improvement in limonene yield suggests that the MsLS enzyme activity was limited by the substrate availability in the V11 strain, which lacks the IUP. Our results are consistent with those of Lin et al. [28], who engineered the MEP pathway to improve the C5 hemiterpene titer of the host and thereby increased the limonene production.

### 2.4. Limonene Production Optimization

**Figure 5:**
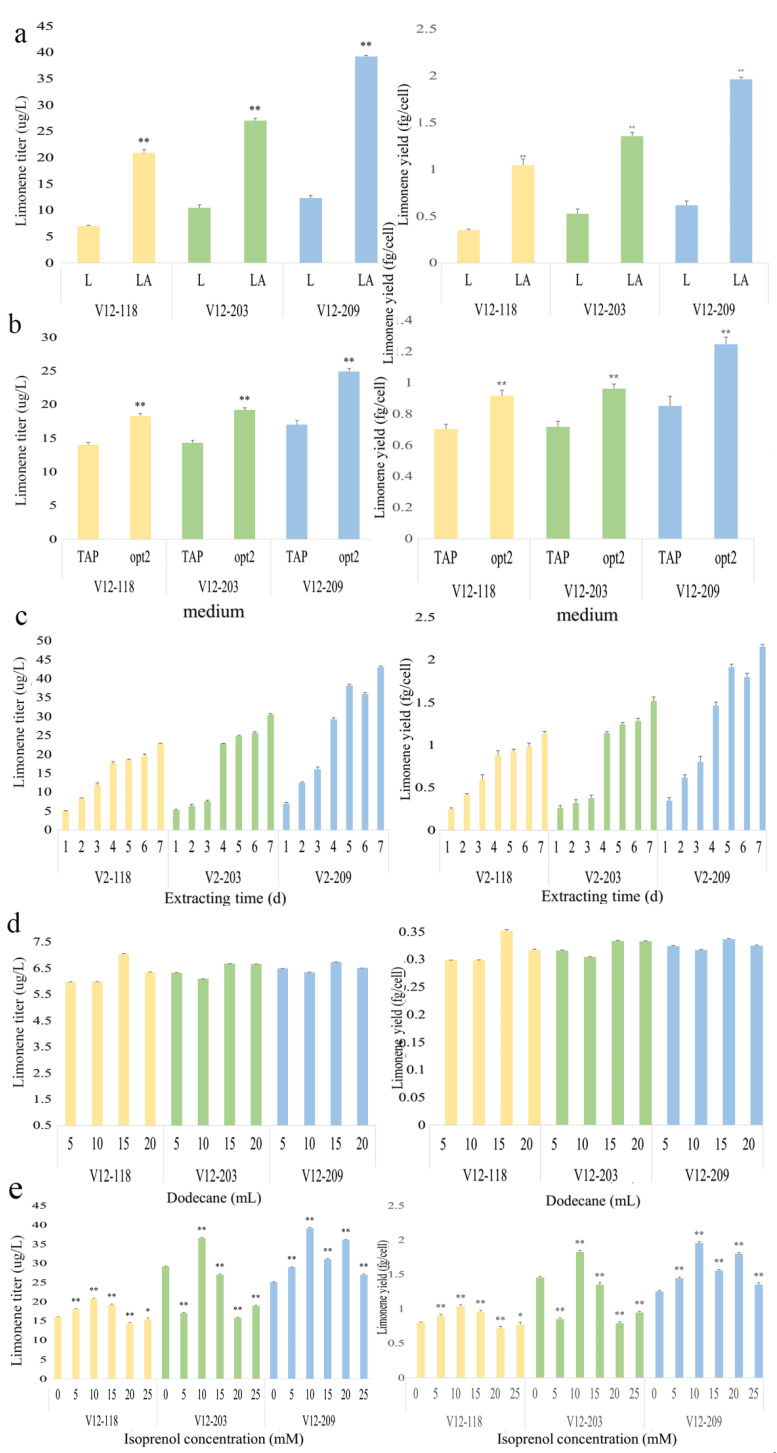
Limonene yield in transgenic algae. (**a**) Strains were grown under constant (24 h) light (L) or 16:8 h light: dark cycles (LA) conditions. Strains were cultivated in 500 mL shake flasks containing a 300 mL TAP medium supplemented with 15 mM isoprenol at 25 °C and 120 rpm under constant (24 h) light or 16:8 h light: dark cycles, and using 5 mL dodecane as overlay for 5 d. (**b**) Strains were cultivated in 500 mL shake flasks containing a 300 mL TAP or opt2 medium supplemented with 15 mM isoprenol at 25°C and 120 rpm under constant (24 h) light, and using 5 mL dodecane as a solvent overlay for 5 d. Strains were cultivated in 500 mL shake flasks containing a 300 mL TAP medium supplemented with 10 mM isoprenol at 25°C and 120 rpm under 16:8 h light: dark conditions using 5 mL dodecane as a extraction overlay for 1, 2, 3, 4, 5, 6 or 7 d (**c**) or using 5, 10, 15 or 20 mL dodecane (**d**) for 7 d. (**e**) Strains were cultivated in 500 mL shake flasks containing a 300 mL TAP medium supplemented with 5, 10, 15, 20 or 25 mM isoprenol at 25 °C and 120 rpm under 16:8 h light: dark conditions, and using 5 mL dodecane as an extraction solvent for 5 d. Three biological replicates were used to calculate the limonene yield averages and SDs. Statistical significance (one-way ANOVA) compared to 0 mM isoprenol is represented by asterisks (** indicates a difference at the *p* ≤ 0.01 level and * indicates a difference at the *p* ≤ 0.05 level).

Photosynthetic microorganisms can grow using solar energy (light), carbon dioxide (CO_2_), or organic carbon sources as the primary inputs to generate terpenoids in a renewable manner [29]. *C. reinhardtii* can undergo photoautotrophic growth in light, heterotrophic growth in darkness on acetate as a sole carbon source, and mixotrophic combinations of these growth modes [30]. In this study, the high-throughput cultivation, using mixotrophic acetate cultivations in a TAP medium (light + acetate) was necessary to compare the genetic constructs used for the limonene production. We carried out a scaled-up cultivation of the highest limonene-producing strains, V12-118, V12-203 and V12-209, in a TAP medium under both constant (24 h) light and 16:8 h light: dark (L:D) cycle conditions. We found that, after 5 d cultivation, the latter conditions, i.e., L:D cycling, gave the highest productivities in all three strains (Figure 5a). The limonene production of the V12-118, V12-203 and V12-209 strains was approximately 21, 27 and 39 µg/L when grown under L:D cycling conditions, respectively, approximately 3-fold more than when the same strains were grown with constant light. This result is in accordance with a previous study of a sesquiterpenoid production, which indicated that L:D regimens promote a slower growth rate and therefore direct more metabolic activity towards the product formation [11]. Thus, L:D cycles can prolong the exponential growth phase, thereby increasing productivity regardless of the carbon regimen.

*C. reinhardtii* can use acetate as the sole carbon source for its heterotrophic growth, but can also use acetate and CO_2_ for its mixotrophic growth. In the absence of CO_2_, acetate will have an important effect on the biomass and heterologous protein production. Song et al. [31] attempted to optimize the growth medium to improve the zeaxanthin yield and found that the increased amounts of acetic acid (opt2 medium) could both promote cell growth and increase the zeaxanthin production in *C. reinhardtii*. We therefore used the opt2 medium with the best limonene-producing strains, V12-118, V12-203 and V12-209, to attempt to increase the limonene production and obtained titers of approximately 18, 19 and 24 µg/L respectively, approximately 1.3-fold the levels achieved after growth in the TAP medium. Thus, compared with the TAP medium, growth in the opt2 medium increased the limonene production significantly in all three strains (Figure 5b), reflecting the experience of Song et al. [31] with zeaxanthin.

To investigate the influence of the dodecane overlay extraction time on the limonene extraction we performed a scaled-up cultivation in a TAP medium of the three best limonene-producing strains, and collected the dodecane fractions after 1, 2, 3, 4, 5, 6 and 7 d growth (Figure 5c). We also investigated whether the volume of dodecane used has an effect, where a 300 mL TAP medium was supplemented with 5, 10, 15 or 20 mL dodecane as an extraction solvent (Figure 5d). We observed the highest limonene accumulation after 7 d, with titers of up to 23, 30 and 43 µg/L from strains V12-118, V12-203 and V12-209, respectively, while a 15-mL overlay of dodecane gave the highest concentration of limonene (approximately 7 µg/mL) in all three strains (Figure 5d). Lauersen et al. [32] investigated the dynamics of the secreted recombinant proteins from *C. reinhardtii* and showed that it was essential to optimize the culture time for the best product yields. Lauersen et al. [9] observed the dynamics of the accumulation of the sesquiterpenoid patchoulol in dodecane and found that the accumulation seemed to continue until the catabolic processes ceased after 144 h. We also found that the limonene accumulation increased in the dodecane fraction even after the algal cells had reached a stationary phase (Figure 5c and Supplementary Figure S2). Davies et al. [26] showed that the dodecane overlay was appropriate for the limonene product capture and did not greatly affect the chlorophyll content or biomass yield of Synechococcus sp. PCC 7002, which was confirmed in our study. The dodecane overlay has also been used in two-phase cultures to capture the heterologous patchoulol [9], diterpenoids [10] and the sesquiterpene biodiesel precursor (E)-α-bisabolene [11] from the cell wall-deficient *C. reinhardtii* UVM4 strain. A 5% dodecane overlay was used in their studies, which is in accordance with our results. The dodecane overlay extraction method probably helps to avoid possible product-induced feedback inhibition, which is often observed in metabolic pathways. Moreover, limonene can kill *C. reinhardtii* cells at a concentration of 0.2 mM [33]; therefore, the dodecane extraction will likely reduce the toxic effect of limonene.

The IUP involves a double phosphorylation (pyrophosphorylation) of the isopentenol substrates, isoprenol or prenol, in two sequential steps in order to produce IPP and DMAPP, the main precursors in the terpenoid synthesis. As mentioned above, the specific concentration of feedstock used is critical for increasing the production of IPP. Thus, we grew the best limonene-producing strains, V12-118, V12-203 and V12-209 in a TAP medium supplemented with 5, 10, 15, 20 or 25 mM isoprenol or prenol to investigate the impact of the different substrates and the substrate concentrations on the limonene production. The results showed that prenol has a lethal effect on the strains, which did not survive at any prenol concentration tested (data not shown); in contrast, isoprenol has a low toxicity and is thus compatible with the *C. reinhardtii* growth. The transformant strains showed the highest limonene production at a 10 mM isoprenol feed concentration, giving limonene productivities in V12-118, V12-203 and V12-209 of approximately 20, 37 and 39 µg/L, respectively (Figure 5e).

## 3. Discussion

*C. reinhardtii* is typical of Chlorophyta in solely relying on the MEP pathway in the chloroplast to supply C5 terpenoid precursors (IPP and DMAPP). Thus, enhancing C5 hemiterpene titers is crucial for producing high-value terpenoids. *C. reinhardtii* chloroplasts can express foreign genes and accumulate heterologous products such as 13R(+) manoyl oxide [10]. However, the position effects and transgene silencing [34] can lead to large variations in the gene expression. As a result, it is usually necessary to screen many transformants to identify a strain with acceptable levels of the transgene expression [35]. Therefore, in this study, we expressed the foreign genes in the nuclear genome of *C. reinhardtii* and targeted the protein products to the algal chloroplast to produce terpenoids.

The V3 and V4 constructs generally showed a higher IPP yield than the V5, V6, V7 and V8 constructs, except that V3 was less productive than V7. Strain V4-49 produced IPP at 2-fold higher levels than strain V7-3 (Figure 3a). This indicates that, the fusion of ScCK and AtIPK in a single polypeptide is possible to express functional products, but the yield of the fusion enzyme is lower than that of the enzyme expressed individually. The expression of consecutive enzymes in a metabolic pathway as the multidomain fusion proteins can lead to a more effective substrate utilization and thereby increase production [9,19,20,21,22], but the nature of the linker peptides used may result in fusion proteins being misfolded, or in a low yield of the multidomain protein, or in a reduced bioactivity [23]. Although the fusion reduces the distance between the respective enzymes, the folding and natural conformation of the multidomain protein can be impaired compared to the free enzymes, and thus can hinder their catalytic ability [24]. The large size of such constructs can also cause false-positive transformants in *C. reinhardtii* [8], which would explain why the IPP yield was lower with the multidomain fusion enzymes.

The limited supply of the precursors is one of the main challenges for the heterologous terpenoid synthesis in microbial hosts [36]. Enhancing the terpenoid titers has often relied on precursor pathway engineering, making both the MEP and MVA pathways critical engineering targets [10]. However, the MEP pathway is highly regulated at the genetic and metabolic levels [37], thus increasing the difficulty of engineering this precursor pathway. Previous studies have engineered the rate-limiting enzymes of the MEP pathway or have used the heterologous expression of the MVA pathway to increase the metabolic flux towards the intermediate metabolites IPP and DMAPP in algae, but with little improvement in the target production [10] or where improvements only emerged after multiple rounds of transformation and selection [38]. For *C. reinhardtii*, the relatively few selectable markers available limit the number of the engineering steps possible [8]; thus, it would be difficult to engineer the seven or six enzymatic steps of the MEP or MVA pathways, respectively, to enhance IPP and DMAPP production in this algae. Recently, an artificial IUP was developed to produce IPP or DMAPP via the sequential phosphorylation of the corresponding alcohol precursors [3,16]. The IUP is much simpler than the natural MVA or MEP pathways, requiring only two reaction steps. It circumvents the intricate regulation required to achieve a high-level accumulation of C5 hemiterpenes via the natural routes and tremendously simplifies the biochemical engineering of the high-value terpenoid production [3,15,16]. However, the IUP needed additional isoprenol, which will increase cost. In this study, we heterologously expressed the key enzymes of the IUP in *C. reinhardtii*, achieving a maximum IPP production of approximately 10.5 mg/L in the V4-49 strain when grown in a TAP medium supplemented with 15 mM isoprenol (Figure 3b). This is approximately 17-fold higher than the yield from UVM4 and demonstrates that the IUP significantly enhances the C5 hemiterpene titer in *C. reinhardtii*.

In this study, the IPP titer was highly improved by the IUP, but the limonene yield was still far lower than other species. Microalgae naturally generate a turnover of isoprenoid products, the FPP synthase (FPPs) catalyzes the sequential condensation of IPP and DMAPP to the FPP (C15), which is the precursor of sterols and ubiquinone (UQ) [9]. The FPPs will compete with GPP synthase (GPPs) to use IPP and DMAPP, which will decrease the IPP and DMAPP flux into GPP. Moreover, microalgae naturally generate a turnover of isoprenoids involved in photosynthesis, e.g., carotenoids and the phytol chain of chlorophyll molecules, which are derived from geranylgeranyl pyrophosphate (GGPP, C20). The GGPP synthase (GGPPs) catalyze the GPP to form the GGPP. The GGPPs will compete with LS in order to use GPP, which will decrease the GPP flux into limonene. What’s more, the low catalytic activity of terpene synthases is another important challenge in addition to the precursor supply [39]. The GPPs and LS should have enough accumulation to outcompete the native metabolism for the use of IPP, DMAPP, and GPP.

Most studies that have attempted to improve the limonene production have focused on increasing the terpene precursor titer [15], for example, by introducing a complete MVA pathway into yeast peroxisomes [40]; overexpressing rate-limiting enzymes of the MEP pathway in cyanobacteria [41]; using neryl diphosphate (cis-isomer of GPP) as a substrate in *S. cerevisiae* and *E. coli* [24]; overexpressing genes in the pentose phosphate (PP) pathway to enhance the C5 hemiterpene levels in the cyanobacterium Synechocystis sp. PCC 6803 [13]; engineering a PDH bypass to enhance the biosynthesis of cytosolic acetyl-CoA in *S. cerevisiae* [5]; or improving the GPP synthesis by a degradative regulation of ERG20 and mutated ERG20WW in *S. cerevisiae* [42]. These results demonstrate that improving the availability of the substrate is an alternative strategy for boosting the limonene production. It is also clear that the strains expressing the IUP can serve as a reliable chassis for a high-value terpenoid production.

**Figure 6:**
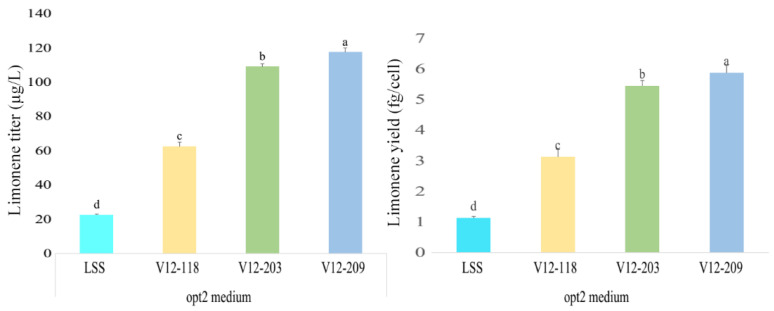
Growth and limonene yield in 300 mL batch cultures of the limonene expression strains under optimized conditions. Strains were cultivated in 500 mL shake flasks containing a 300 mL opt2 medium supplemented with 10 mM isoprenol at 25 °C and 120 rpm under 16:8 h light: dark conditions, and using 15 mL dodecane as the overlay for 7 d. Three biological replicates were used to calculate the limonene yield averages and SDs. One-way ANOVA was used for the statistical analyses. Different letters (a–d) indicate significant differences (*p* ≤ 0.05).

Growing the above strains for 7 d in the opt2 medium supplemented with 10 mM isoprenol and a 15 mL dodecane overlay under a L:D regimen resulted in the highest limonene production levels (Figure 6). Under the optimized fermentation conditions, a maximum limonene content of 117 µg/L was achieved, which is the highest reported to date in *C. reinhardtii*. However, this yield was still far lower than that of Lin and colleagues [28], which was obtained by engineering the fast-growing cyanobacterium Synechococcus elongatus UTEX 2973. Compared with *C. reinhardtii*, the cyanobacteria have an ease of overexpression, have more reliable genetic tools available, and have a higher growth rate and biomass, which may be the reason for the limonene yield gap. Compared with other studies, by only improving the IPP production may not be enough to significantly increase the limonene production. To further improve the production of limonene, we should improve GPP and/or NPP synthesis which will produce more limonene precursor GPP or NPP for producing limonene. Microalgae naturally generate a significant turnover of isoprenoid products, such as sterols and UQ [9] and pigments associated with their color and photosynthetic machinery [4]. Sterols and UQ are derived from FPP (C15), and isoprenoids involved in photosynthesis, e.g., carotenoids and the phytol chain of chlorophyll molecules are derived from GGPP, (C20). Taking this into account, we should decrease the metabolic flux of the compete pathway of GPP flowed to FPP to produce sesquiterpenes or GGPP to produce the photosynthetically associated isoprenoid products that are more beneficial for the GPP supply and the limonene biosynthesis. In addition, the titer of the limonene synthase may not yet be sufficient to exhaust the freely available GPP pool to produce limonene, for future study, we should look at the over expression of limonene synthase proteins, involving either high copy plasmids or multiple copies of a transgene expression cassette introduced into the genome for improving the limonene production.

The lycopene production using the IUP is on par with or exceeds the reported production in engineered strains of *E. coli* that use the MVA pathway, which means the expression of the IUP can produce a sufficient IPP accumulation [16]. This study demonstrates that green algae can use the IUP to heterologously produce limonene. The IUP comprises only two reaction steps, and therefore combining the IUP with a downstream product-forming pathway should be much simpler and thereby should facilitate the metabolic engineering for enhancing the isoprenoid titers. Eukaryotic microalgae, such as *C. reinhardtii*, are interesting candidates for the development of sustainable light-driven bioprocesses, especially the production of heterologous isoprenoid products. They share the evolutionary ancestry with land plants, and therefore it is possible that the microalgal cellular environment is more favorable to the plant terpene synthases (TPSs) than bacterial, yeast or cyanobacterial hosts [8]. Further studies will focus on the optimization of the combination of the IUP with the downstream product-forming pathways in eukaryotic microalgae or plants in order to produce high-value terpenoids.

## 4. Materials and Methods

### 4.1. C. reinhardtii Strain and Cultivation Conditions

The *C. reinhardtii* UV-mutated 4 (UVM4, cell wall-deficient) strain, which is capable of efficiently expressing transgenes and overcomes the long-standing obstacle of the disappointingly poor expression of transgenes in the algal nuclear genome [43], was used for all of the experiments in this work. This algal species originated from Prof. Dr. Ralph Bock and can grow well in freshwater environments. Unless otherwise noted, strain UVM4 was cultured in a Tris-acetate-phosphate (TAP) medium [44] on agar plates or in liquid culture with continuous light (150 μmol m^−2^ s^−1^) at 25 °C. The TAP plates supplemented with antibiotics as appropriate (8 mg L^−1^ paromomycin, 9 mg L^−1^ hygromycin, 10 mg L^−1^ zeocin, and/or 200 mg L^−1^ spectinomycin) were used to maintain the transformants. Microtiter plates or Erlenmeyer flasks containing TAP without antibiotics were used for the liquid cultures.

### 4.2. Plasmid Construction, Transformation, and Screening of Mutants

All of the cloning in this work was performed with Thermo Fisher Scientific FastDigest restriction enzymes (Thermo Fisher Scientific, Shanghai, China) and the ClonExpress^®^ II One Step Cloning Kit (Vazyme, Nanjing, China), according to the manufacturers’ protocols. All PCRs were performed using the KOD OneTM PCR Master Mix -Blue- (TOYOBO, Shanghai, China), following the manufacturer’s protocols, and the primers are listed in Supplementary Table S1. Following each cloning step, the vector sequences were confirmed by sequencing (Sangon Biotech, Shanghai, China). The plasmids were transformed into *Escherichia coli* Top10, and the transformants were cultivated in Luria broth (LB) agar plates or in liquid medium with 100 mg L^−1^ ampicillin.

The amino acid sequences for the choline kinase from *Saccharomyces cerevisiae* (ScCK, AAA34499.1), isopentenyl phosphate kinase from *Arabidopsis thaliana* (AtIPK, AAN12957.1), isopentenyl-pyrophosphate delta isomerase from *E. coli* (IDI, AAD26812.1) and 4S-limonene synthase from *Mentha spicata* (MsLS, AAC37366.1) were codon optimized and synthesized de novo taking account of the nuclear codon bias of *C. reinhardtii* (General Biosystem Company, Anhui, China). The MsLS was optimized for codon usage in *C. reinhardtii* without the predicted chloroplast transit peptide (cTP) and is referred to in this work as MsLS. To minimize the exon lengths and improve the overall expression, the first intron of ribulose bisphosphate carboxylase small subunit 2 (rbcS2i1) was added to the synthetic transgene sequence, and the second intron (rbcS2i2) was incorporated into the last position between two guanine nucleotides (G/intron/G) [45]. All codon-optimized, intron-containing nucleotide sequences are listed in Supplementary Table S2 for *ScCK* (GenBank accession no. OP046413), *AtIPK* (GenBank accession no. OP046414), *IDI* (GenBank accession no. OP046415), and *MsLS* (GenBank accession no. OP046416). The sequences mentioned above were designed with the compatible restriction endonuclease sites and cloned into pOptimized (pOpt) Chlamydomonas expression vectors [46] in the fusion with either mVenus (yellow), mCerulean3 (cyan) or mRuby2 (red) fluorescent protein (Y/C/RFP) reporters and the selection markers as indicated. The pOpt vectors were modified to contain a 36-amino-acid photosystem I reaction center subunit II (PsaD) target peptide and thus can directly target the expressed proteins to the algal chloroplast. All constructs created are listed in Table 1. The key construct maps were shown in Supplementary Figure S3.

The plasmids were introduced into *C. reinhardtii* by glass bead agitation [47]. The positive transformants were selected on TAP agar plates supplemented with suitable antibiotics at 8 mg L^−1^ (paromomycin), 9 mg L^−1^ (hygromycin B), 10 mg L^−1^ (zeocin) or 200 mg L^−1^ (spectinomycin) at a light intensity of 150 µmol photons m^−2^ s^−1^. The colonies were maintained on TAP agar plates with the respective antibiotic combinations. The colonies exhibiting fluorescence were picked for the TAP medium and cultivated with 150 µmol photons m^−2^ s^−1^ light intensity at 120 rpm until sufficiently dense (mid-logarithmic phase or stationary phase). As Lauersen et al. [46] reported, for the YFP, mVenus, the excitation was at 514 nm and the emission was between 520 and 550 nm; for the CFP, mCerulean3, the excitation was at 458 nm and the emission was between 460 and 490 nm; and for the RFP, mRuby2, the excitation was at 561 nm and the emission was between 590 and 620 nm. A microplate reader (Varioskan Flash, Thermo Scientific, USA) was used to detect the YFP, CFP and RFP fluorescence signals to verify the expression of the target constructs. The original fluorescence abundances were corrected with the cell density to make the data comparable. The fluorescence imaging was carried out using a confocal laser microscope with respective filter sets and the excitation and emission wavelengths previously described for each reporter (LEICA-TCS SP8(A7-345), Leica, Germany). The transformants were further confirmed by the genomic PCR and qRT-PCR methods (Supplementary Figure S4). The expression of the ScCK, AtIPK, IDI, and MsLS reporter fusions to full length was confirmed via SDS PAGE and western blotting using α-StrepII tag-HRP linked antibody (IBA Lifesciences).

### 4.3. Extraction and Analysis of the C5 Hemiterpene Diphosphates

The C5 hemiterpene diphosphate yield was tested for each genetic construct with three representative strains, each in biological triplicate. For the cultivation, shake flasks with a 150 mL TAP medium supplemented with the specified concentration of isoprenol or prenol were shaken at 120 rpm, 25 °C and 150 µmol photons m^−2^ s^−1^ continuous light until the stationary phase. To harvest the cultures, cells were centrifugated at 3000 rcf for 10 min at 4 °C. The cells were resuspended in a 5 mL ice-cold mixture of water/methanol (1:1; *v*/*v*), frozen at −80 °C and then thawed at 4 °C. The freeze-thaw process was repeated three times until the cells were completely lysed. The crude extract was centrifuged at 8000 rcf for 10 min at 4 °C. The supernatant was combined and filtered through a filter film (0.22 µm) for the following LC-MS/MS analysis.

The LC-MS/MS assay was performed on a Series 6120 system (Agilent Technologies, Santa Clara, CA, USA). The C5 hemiterpene diphosphates were separated at a flow rate of 0.2 mL/min on a Phenomenex Gemini-NX C18 column (5 µm, 150 × 2.0 mm, Phenomenex Inc., Aschaffenburg, Germany) using solution A (5 mM NH_4_HCO_3_ in water) and solution B (100% acetonitrile). The gradient elution was 90% A, 10% B at 0 min, followed by the linear gradient to 10% A, 90% B at 8 min, maintained at 10% A, 90% B to 16 min, then returned to the initial conditions by 16.1 min, and then re-equilibrated at the initial conditions by 25 min. The injection volume was 2 µL. The column temperature was maintained at 35 °C. An IPP standard (Sigma-Aldrich, Inc., St. Louis, MO, USA) was used to identify the target in the extract. The mass spectrometric data were recorded in MRM mode with negative ionization using parameters: *m/z* (Q1): 245; *m/z* (Q3): 79. To determine the concentration of IPP in the testing sample, a calibration curve was constructed using a series of plots with 0.5, 1, 2, 3, 4 and 5 µM IPP standards (Supplementary Figure S5). IPP was detected in the HPLC-MS/MS chromatograms of the extraction agent (water/methanol (1:1; *v*/*v*)) and exhibited an appropriate mass fractionation pattern compared to the pure standard (Supplementary Figure S6). All organic solvents (acetonitrile and methanol) used in this study were of HPLC grade.

### 4.4. Extraction and Analysis of Limonene

The assessment of limonene yield was conducted for each vector construct with three representative strains, each in biological triplicate. The strains were inoculated in a 300 mL TAP medium supplemented with isoprenol or prenol at the desired concentration. Following the addition of the 5 mL dodecane overlay, the strains were shaken at 120 rpm, 25 °C, with 150 µmol photons m^−2^ s^−1^ continuous light for 5 d. For the sampling, the dodecane fractions were collected by pipette removal from the culture, followed by centrifugation at 8000 rcf for 6 min, and then transferred to new sample tubes.

Following the filtration, the quantification of limonene in dodecane was carried out by gas chromatography-mass spectroscopy (GC-MS) (Hewlett-Packard model 7890 A, Agilent Technologies, Santa Clara, CA, USA), equipped with an Rxi-5MS column (30 m × 0.25 mm × 0.25 µm, Restek, Alexandria LA, USA). Helium was used as a carrier gas and the injection volume was set to 2 µL (80 °C, 2 min; 5 °C/min to 90 °C, hold 2 min; 20 °C/min to 280 °C, hold 2 min). The retention time of limonene was confirmed using the commercial standard (*R*)-limonene (Merck KGaA, Darmstadt, Germany) [48]. The quantification of limonene was performed using a standard curve of the commercial (*R*)-limonene. The standard calibration curves in the range of 0.5–5 ppm limonene in dodecane were used to quantify the amount of limonene, and the R2 coefficient for the calibration was greater than 0.99 (standard curve in Supplementary Figure S7). The temperature in the gas chromatograph-mass transfer line was 250 °C, and the mass spectrometer was set to full-scan mode. The extracted-ion chromatograms (XIC) with mass ranges of 68, 93, 121 and 136 for was used for the limonene determination. All measurements were performed in triplicate and the chromatograms were reviewed manually.

### 4.5. Optimization of the Fermentation and Limonene Extraction Conditions

The three highest-producing limonene strains were subjected to the optimized fermentation and limonene extraction conditions in order to enhance limonene production. The strains were cultured under constant (24 h) light or 16:8 h light: dark cycle conditions to determine the influence of the light regimen on the limonene production; in a TAP medium or opt2 medium (TAP medium + 1 mL L^–1^ glacial acetic acid) to determine which of the two was best for the limonene production; and then stirred in 500 mL flasks containing a 300 mL TAP medium supplemented with 5, 10, 15, 20 and 25 mM isoprenol to identify the effect of the feed substrate and concentration. The dodecane solvent overlay can extract limonene from algal cells; thus, we investigated the influence of the different dodecane extraction times (1, 2, 3, 4, 5, 6 and 7 d) and dodecane volumes (5, 10, 15 and 20 mL) on the limonene production. All the fermentation processes were performed in triplicate. At the end of the fermentation, the dodecane fractions were collected and assayed.

### 4.6. Statistical Analysis

All of the measurements were performed in triplicate. The results are presented as the mean + standard deviation (SD). One-way ANOVA was used to analyze the significance of the differences, and the statistical significance was indicated by *p* < 0.05 or *p* < 0.01.

## 5. Conclusions

Here, we show that *C. reinhardtii* can use the IUP to produce two sequential phosphorylations of isopentenol substrates, isoprenol or prenol, to provide sufficient IPP or DMAPP for the heterologous production of limonene. This is preferable to the use of other pathways, such as the MEP pathway, where there are major issues involving the restricted carbon flux. Furthermore, the IUP is much simpler than the natural MVA or MEP pathways, and requires only two reaction steps, which should facilitate future engineering attempts at producing high-value terpenoids. A high-level C5 hemiterpene diphosphate-producing background strain can be used as a chassis to produce high-value terpenoids without multiple engineering steps. The ability to utilize the IUP in photosynthetic microorganisms will allow synthetic biologists to make substantial improvements in the isoprenoid metabolism and produce a diverse array of terpenoid products. The results also demonstrate that the modification of the cultivation and extraction parameters can greatly improve the product outcome. Our work provides a meaningful new strategy for the combinatorial metabolic engineering of *C. reinhardtii* for the efficient production of terpenoids.

## Figures and Tables

**Table 1:** Genetic constructs used in this study.

Construct Name	Vector	Antibiotic Resistance in *C. reinhardtii*	Source
pOpt_CTP_mVenus_Hyg	V1	hygromycin B	This study
pOpt_CTP_mCerulean3_Paro	V2	paromomycin	This study
YFP_ScCK	V3	hygromycin B	This study
CFP_AtIPK		paromomycin	
ScCK_YFP	V4	hygromycin B	This study
AtIPK_CFP		paromomycin	
ScCK_AtIPK_YFP	V5	hygromycin B	This study
AtIPK_ScCK_YFP	V6	hygromycin B	This study
ScCK_YFP_AtIPK	V7	hygromycin B	This study
AtIPK_CFP_ScCK	V8	paromomycin	This study
pOpt_CTP_mRuby2_Ble	V9	zeocin	This study
pOpt_CTP_aadA	V10	spectinomycin	This study
MsLS_aadA	V11	spectinomycin	This study
MsLS_aadA	V12	spectinomycin	This study
IDI_RFP		zeocin	

## Data Availability

Not applicable.

## Supplementary Materials

The following supporting information can be downloaded at: https://www.mdpi.com/article/10.3390/md20090577/s1, Figure S1: Investigating the influence of the different concentrations of prenol or isoprenol on algae growth. Three biological replicates were used to calculate the gene relative expression averages and standard deviations (SD) for all measures. Statistical significance (one-way ANOVA) compared to UVM4 is represented by asterisks (** indicates a difference at the *p* ≤ 0.01 level and * indicates a difference at the *p* ≤ 0.05 level); Figure S2: Daily samples were taken to analyze growth (OD750) during the cultivation of three transgenic algae; Figure S3: The construct maps used in this study. Figure S4: Relative transcription level of the genes in transgenic algae. Three biological replicates were used to calculate the gene relative expression averages and standard deviations (SD) for all measures. Statistical significance (one-way ANOVA) compared to UVM4 is represented by asterisks (** indicates a difference at the *p* ≤ 0.01 level and * indicates a difference at the *p* ≤ 0.05 level); Figure S5: IPP standard curve; Figure S6: IPP could be detected readily by LC-MS/MS in the extraction agent from the cultivated algal cells; Figure S7: Limonene standard curve; Table S1: List of primers used in this study; Table S2: List of codon-optimized, intron-containing nucleotide sequences of *ScCK*, *AtIPK*, *IDI*, and *MsLS*.

## Abbreviations

ADH: alcohol-dependent hemiterpene; *C. reinhardtii*: *Chlamydomonas reinhardtii*; CFP: mCerulean3 (cyan) fluorescent protein; CK: choline kinase; C5: five-carbon; DMAPP: dimethylallyl diphosphate; GC-MS: gas chromatography-mass spectroscopy; IDI: diphosphate isomerase; IPK: isopentenyl phosphate kinase; IPP: isopentenyl pyrophosphate; IUP: isopentenol utilization pathway; L:D: light: dark; LS: limonene synthase; MEP: 2-C-methyl-D-erythritol 4-phosphate; MVA: mevalonate; pOpt: pOptimized; RFP: mRuby2 (red) fluorescent protein; TAP: Tris-acetate-phosphate; UVM4: *C. reinhardtii*; UV-mutated 4; YFP: mVenus (yellow) fluorescent protein.
