# Supplementary material for: Metabolic Engineering of the Isopentenol Utilization Pathway Enhanced the Production of Terpenoids in Chlamydomonas reinhardtii
Source: Mar Drugs. 2022 Sep 15;20(9):577. doi: 10.3390/md20090577 (PMC9505001; doi:10.3390/md20090577)
Supplement: Supplementary file 1 [file marinedrugs-20-00577-s001.zip › Table S2.pdf]

**Table S2.** List of codon-optimized, intron-containing nucleotide sequences of *ScCK*, *AtIPK*, *IDI*, and *MsLS*.

***ScCK***

ATGGTACAAGAGTCCCGCCCCGGCTCCGTCCGCTCCTACTCCGTCGGATAC
CAGgtgagtcgacgagcaagcccggcggatcaggcagcgtgcttgcagatttgacttgcaacgcccgcattgtgtcga
cgaaggcttttggctcctctgtcgctgtctcaagcagcatctaaccctgcgtcgccgtttccatttgcagGCCCGCTCG
CGCTCCTCCTCTCAACGAAGACACTCCCTGACCCGCCAGCGAAGCAGCCA
GCGCCTGATCCGCACCATCAGCATCGAGAGCGACGTCAGCAACATCACCG
ACGACGACGACCTGCGCGCCGTGAACGAGGGCGTGGCCGGCGTGCAACT
GGACGTCAGCGAGACCGCCAACAAGGGCCCCCGCCGCGCCAGCGCCACC
GACGTGACCGATAGCCTGGGAAGCACCAGTAGCGAGTACATCGAGATCCC
CTTCGTGAAGGAGACCCTGGACGCCAGCCTGCCAAGCGACTACCTGAAGC
AAGACATCCTGAACCTGATCCAGAGCCTGAAGATCAGCAAGTGGTACAAC
AACAAGAAGATCCAGCCCGTGGCCCAAGACATGAACCTAGTGAAGATCAG
CGgtgagtcgacgagcaagcccggcggatcaggcagcgtgcttgcagatttgacttgcaacgcccgcattgtgtcgacg
aaggcttttggctcctctgtcgctgtctcaagcagcatctaaccctgcgtcgccgtttccatttgcagGCGCCATGAC
CAACGCCATCTTCAAGGTGGAGTACCCCAAGCTGCCAAGCCTGCTGCTGC
GCATCTACGGCCCCAACATCGACAACATCATCGACCGCGAATACGAGCTGC
AAATCCTGGCCCGCCTGAGCCTGAAGAACATCGGACCCAGCCTATACGGCT
GCTTCGTGAACGGCCGCTTCGAACAGTTCCTGGAGAACAGCAAGACCCTG
ACCAAGGACGACATCCGCAACTGGAAGAACAGCCAGCGCATCGCCCGCCG
CATGAAGGAGCTGCACGTCGGCGTGCCCCTGCTGAGTAGCGAGCGCAAGA
ACGGAAGCGCCTGCTGGCAAAAGATCAACCAGTGGCTGCGCACCATCGAG
AAAGTGGACCAATGGGTTGgtgagtcgacgagcaagcccggcggatcaggcagcgtgcttgcagattt
gacttgcaacgcccgcattgtgtcgacgaaggcttttggctcctctgtcgctgtctcaagcagcatctaaccctgcgtcgccg
tttccatttgcagGCGACCCCAAGAACATCGAGAACAGCCTGCTATGCGAGAACTG
GAGCAAGTTCATGGACATCGTGGACCGCTACCACAAGTGGCTGATAAGCC
AGGAGCAAGGCATCGAGCAAGTGAACAAGAACCTGATCTTCTGCCACAAC
GACGCCCAATACGGCAACCTGCTGTTCACCGCCCCCGTGATGAACACCCCA
AGCCTGTACACCGCCCCAAGTAGCACCAGCCTGACCAGCCAAAGCAGTAG
CCTGTTCCCAAGTAGTAGCAACGTGATCGTGGACGACATCATCAACCCCCC
CAAGCAAGAGCAGAGCCAGGACAGCAAGCTGGTAGTGATCGACTTCGAAT
ACGCCGGCGCCAACCCCGCCGCCTACGACCTGGCCAACCACCTGAGCGAG
TGGATGTACGACTACAACAACGCCAAGgtgagtcgacgagcaagcccggcggatcaggcagc
gtgcttgcagatttgacttgcaacgcccgcattgtgtcgacgaaggcttttggctcctctgtcgctgtctcaagcagcatctaa
ccctgcgtcgccgtttccatttgcagGCCCCCCACCAGTGCCACGCCGACCGCTACCCCGA
CAAGGAGCAAGTGCTGAACTTCCTGTATAGCTACGTCAGCCACCTGCGCGG
CGGCGCCAAGGAGCCCATCGACGAGGAAGTGCAACGCCTGTACAAGAGC
ATAATCCAGTGGCGCCCCACCGTGCAACTGTTCTGGAGCCTATGGGCCATC
CTGCAAAGCGGAAAGCTGGAGAAGAAGGAGGCCAGCACCGCCATCACCC
GCGAGGAGATCGGCCCCAACGGAAAGAAGTACATCATCAAGACCGAGCCC
GAGAGCCCCGAGGAGGACTTCGTGGAGAACGACGAGCCCGAGGCCG
GCGTCAGCATCGACACCTTCGACTACATGGCCTACGGCCGCGACAAGATCG
CCGTGTTCTGgtgagcttgcggggttgcgagcaacactccagcaacgaacagtgcccaagtcaggaatctgcag

tcagcctgggctttcggcggctttttcttgggcaaacagcttgcactcatgccagcgcggcttgtccagcctcacttgagcttt
ccagctgctaccagccgggctatacgacagcgacagagccatagcgtggaatcacttatttgggttgccgaagtagcggt
cggagcgtgagttcttggtcaagccgccccttatccggttcctgtccgtgtctttgtccctcgttcacccttcgcggcacccttc
atccccttgcttgcagGGGCGACCTGATCGGCCTGGGCATCATCACCGAGGAGGAGT
GCAAGAACTTCAGTAGCTTCAAGTTCCTGGACACCAGCTACCTGtaaGCGG

***At**IPK***

ATGGAGCTTAACATCTCCGAGTCGCGTTCCCGAAGCATTCGCTGCATTGTC
AAGCTGGGAGGCGCCGCCATCACCTGCAAGAACGAGCTGgtgagtcgacgagcaag
cccggcggatcaggcagcgtgcttgcagatttgacttgcaacgcccgcattgtgtcgacgaaggcttttggctcctctgtcg
ctgtctcaagcagcatctaaccctgcgtcgccgtttccatttgcagGAGAAGATCCACGACGAGAACC
TGGAGGTGGTGGCCTGCCAGCTGCGCCAGGCCATGCTGGAAGGCAGCGCC
CCAAGCAAAGTGATCGGCATGGACTGGAGCAAGCGCCCCGGAAGCAGCG
AGATCAGCTGCGACGTGGACGACATCGGCGACCAGAAAAGCAGCGAGTTC
AGCAAGTTCGTGGTAGTGCACGGCGCCGGAAGCTTCGGCCACTTCCAAGC
CAGCCGAAGCGGCGTGCACAAGGGCGGCCTGGAGAAGCCCATCGTGAAG
GCCGGCTTCGTGGCCACCCGCATCAGCGTGACCAACCTGAACCTGGAGAT
CGTCCGCGCCCTGGCCCGCGAGGGCATCCCCACCATCGGCATGAGCCCCTT
TAGCTGCGGCTGGAGCACCAGCAAGCGCGACGTGgtgagtcgacgagcaagcccggcg
gatcaggcagcgtgcttgcagatttgacttgcaacgcccgcattgtgtcgacgaaggcttttggctcctctgtcgctgtctcaa
gcagcatctaaccctgcgtcgccgtttccatttgcagGCCAGCGCCGACCTGGCCACCGTGGCCA
AGACCATAGACAGCGGCTTCGTGCCCGTGCTGCACGGCGACGCCGTGCTG
GACAACATCCTGGGCTGCACCATCCTGAGCGGCGACGTGATCATCCGCCAC
CTGGCCGACCACCTGAAGCCCGAATACGTGGTGTTCCTGACCGACGTGCTG
GGCGTGTACGACCGCCCCCCAAGCCCAAGCGAGCCCGACGCCGTGCTGCT
GAAGGAGATCGCCGTCGGAGAGGACGGAAGCTGGAAGGTAGTGAACCCC
CTGCTGGAGCACACCGACAAGAAAGTGGACTATAGCGTGGCCGCCCACGA
CACCACCGGCGGCATGGAGACCAAGATCAGCGAGGCCGCCATGATCGCCA
AGCTGGGCGTGGACGTGTACATCGTGAAGgtgagcttgcggggttgcgagcaacactccagca
acgaacagtgcccaagtcaggaatctgcagtcagcctgggctttcggcggctttttcttgggcaaacagcttgcactcatgc
cagcgcggcttgtccagcctcacttgagctttccagctgctaccagccgggctatacgacagcgacagagccatagcgtg
gaatcacttatttgggttgccgaagtagcggtcggagcgtgagttcttggtcaagccgccccttatccggttcctgtccgtgtc
tttgtccctcgttcacccttcgcggcacccttcatccccttgcttgcagGCCGCCACCACACATAGCCAGC
GCGCCCTGAACGGCGACCTGCGCGACAGCGTGCCCGAGGACTGGCTGGGC
ACCATCATCCGATTCAGCAAGtaaGCGG

***IDI***

ATGCAGACCGAGCACGTGATCCTGCTGAACGCCCAAGGAGTGCCCACCGG
AACGTTGGAGAAATACGCCGCCCACACCGCGGACACCCGGCTGCACCTGgt
gagtcgacgagcaagcccggcggatcaggcagcgtgcttgcagatttgacttgcaacgcccgcattgtgtcgacgaagg
cttttggctcctctgtcgctgtctcaagcagcatctaaccctgcgtcgccgtttccatttgcagGCCTTTAGTAGCT
GGCTGTTCAACGCCAAGGGCCAGCTGCTAGTGACCCGCCGCGCCCTGAGC
AAGAAGGCCTGGCCCGGCGTGTGGACAAACAGCGTATGCGGCCACCCCCA
ACTGGGAGAGAGCAACGAGGACGCCGTGATCCGCCGCTGCCGCTACGAGC

TGGGCGTGGAGATCACCCCCCCCGAGAGCATCTACCCCGACTTCCGCTACC
GCGCCACCGACCCAAGCGGCATCGTGGAGAACGAGGTCTGCCCCGTGTTC
GCCGCCCGCACCACCAGCGCCCTGCAAATCAACGACGACGAGGTGATGGA
CTACCAGTGGTGTGACCTGGCCGACGTGCTGCACGgtgagcttgcggggttgcgagcaa
cactccagcaacgaacagtgcccaagtcaggaatctgcagtcagcctgggctttcggcggctttttcttgggcaaacagctt
gcactcatgccagcgcggcttgtccagcctcacttgagctttccagctgctaccagccgggctatacgacagcgacagag
ccatagcgtggaatcacttatttgggttgccgaagtagcggtcggagcgtgagttcttggtcaagccgccccttatccggttc
ctgtccgtgtctttgtccctcgttcacccttcgcggcacccttcatccccttgcttgcagGCATCGACGCCACCC
CCTGGGCCTTTAGCCCCTGGATGGTGATGCAAGCCACCAACCGCGAGGCCC
GCAAGCGCCTGAGCGCCTTCACCCAACTGAAGtaaGCGG

***Ms**LS*

ATGGAGTTCCGCGTGCACCTGCAGGCCGACAACGAGCAGAAGATTTTCCA
GAACCAGATGAAGCCCGAGCCCGAGgtgagtcgacgagcaagcccggcggatcaggcagcgtg
cttgcagatttgacttgcaacgcccgcattgtgtcgacgaaggcttttggctcctctgtcgctgtctcaagcagcatctaaccc
tgcgtcgccgtttccatttgcagGCCAGCTACCTGATCAACCAGCGCCGCAGCGCCAACT
ACAAGCCCAACATCTGGAAGAACGACTTCCTGGACCAGAGCCTGATCAGC
AAGTACGACGGCGACGAGTACCGCAAGCTGAGCGAGAAGCTGATCGAGG
AGGTGAAGATTTACATCAGCGCCGAGACGATGGACCTGGTGGCCAAGCTG
GAGCTGATCGACAGCGTGCGCAAGCTGGGCCTGGCCAACCTGTTCGAGAA
GGAGATCAAGGAGGCCCTGGACAGCATCGCCGCCATCGAGAGCGACAACC
TGGGCACCCGCGACGACCTGTACGGCACCGCCCTGCACTTCAAGATCCTGC
GCCAGCACGGCTACAAGGTGAGCCAGGACATCTTCGGCCGCTTCATGGAC
GAGAAGgtgagtcgacgagcaagcccggcggatcaggcagcgtgcttgcagatttgacttgcaacgcccgcattgt
gtcgacgaaggcttttggctcctctgtcgctgtctcaagcagcatctaaccctgcgtcgccgtttccatttgcagGGCAC
CCTGGAGAACCACCACTTCGCCCACCTGAAGGGCATGCTGGAGCTGTTCG
AGGCCAGCAACCTGGGCTTCGAGGGCGAGGACATCCTGGACGAGGCCAA
GGCCAGCCTGACCCTGGCCCTGCGCGACAGCGGCCACATCTGCTACCCCG
ACAGCAACCTGAGCCGCGACGTGGTGCACAGCCTGGAGCTGCCCAGCCAC
CGCCGCGTGCAGTGGTTCGACGTGAAGTGGCAGATCAACGCCTACGAGAA
GGACATCTGCCGCGTGAACGCCACCCTGCTGGAGCTGGCCAAGCTGAACT
TCAACGTGGTGCAGGCCCAGCTGCAGAAGAACCTGCGCGAGGCCAGCCG
CTGGTGGGCCAACCTGGgtgagtcgacgagcaagcccggcggatcaggcagcgtgcttgcagatttgact
tgcaacgcccgcattgtgtcgacgaaggcttttggctcctctgtcgctgtctcaagcagcatctaaccctgcgtcgccgtttcc
atttgcagGCATCGCCGACAACCTGAAGTTCGCCCGCGACCGCCTGGTGGAGT
GCTTCGCCTGCGCCGTGGGCGTGGCCTTCGAGCCCGAGCACAGCAGCTTC
CGCATCTGCCTGACCAAGGTGATCAACCTGGTGCTGATCATCGACGACGTG
TACGACATCTACGGCAGCGAGGAGGAGCTGAAGCACTTCACCAACGCCGT
GGACCGCTGGGACAGCCGCGAGACGGAGCAGCTGCCCGAGTGCATGAAG
ATGTGCTTCCAGGTGCTGTACAACACCACCTGCGAGATCGCCCGCGAGATC
GAGGAGGAGAACGGCTGGAACCAGGTGCTGCCCCAGCTGACCAAGGTGT
GGGCCGACTTCTGCAAGGCCCTGCTGGTGGAGGCCGAGTGGTACAACAAG
AGCCACATCCCCACCCTGgtgagtcgacgagcaagcccggcggatcaggcagcgtgcttgcagatttga
cttgcaacgcccgcattgtgtcgacgaaggcttttggctcctctgtcgctgtctcaagcagcatctaaccctgcgtcgccgttt

ccatttgcagGAGGAGTACCTGCGCAACGGCTGCATCAGCAGCAGCGTGAGCGT
GCTGCTGGTGCACAGCTTCTTCAGCATCACCCACGAGGGCACCAAGGAGA
TGGCCGACTTCCTGCACAAGAACGAGGACCTGCTGTACAACATCAGCCTG
ATCGTGCGCCTGAACAACGACCTGGGCACCAGCGCCGCCGAGCAGGAGCG
CGGCGACAGCCCCAGCAGCATCGTGTGCTACATGCGCGAGGTGAACGCCA
GCGAGGAGACGGCCCGCAAGAACATCAAGGGCATGATCGACAACGCCTGG
AAGAAGGTGAACGGCAAGTGCTTCACCACCAACCAGGTGCCCTTCCTGAG
CAGCTTCATGAACAACGCCACCAACATGGCCCGCGTGgtgagcttgcggggttgcgag
caacactccagcaacgaacagtgcccaagtcaggaatctgcagtcagcctgggctttcggcggctttttcttgggcaaaca
gcttgcactcatgccagcgcggcttgtccagcctcacttgagctttccagctgctaccagccgggctatacgacagcgaca
gagccatagcgtggaatcacttatttgggttgccgaagtagcggtcggagcgtgagttcttggtcaagccgccccttatccg
gttcctgtccgtgtctttgtccctcgttcacccttcgcggcacccttcatccccttgcttgcagGCCCACAGCCTGT
ACAAGGACGGCGACGGCTTCGGCGACCAGGAGAAGGGCCCCCGCACCCA
CATCCTGAGCCTGCTGTTCCAGCCCCTGGTGAACTAA
