# Supplementary figures and images for: Metabolic Engineering of the Isopentenol Utilization Pathway Enhanced the Production of Terpenoids in Chlamydomonas reinhardtii
Source: Mar Drugs. 2022 Sep 15;20(9):577. doi: 10.3390/md20090577 (PMC9505001; doi:10.3390/md20090577)

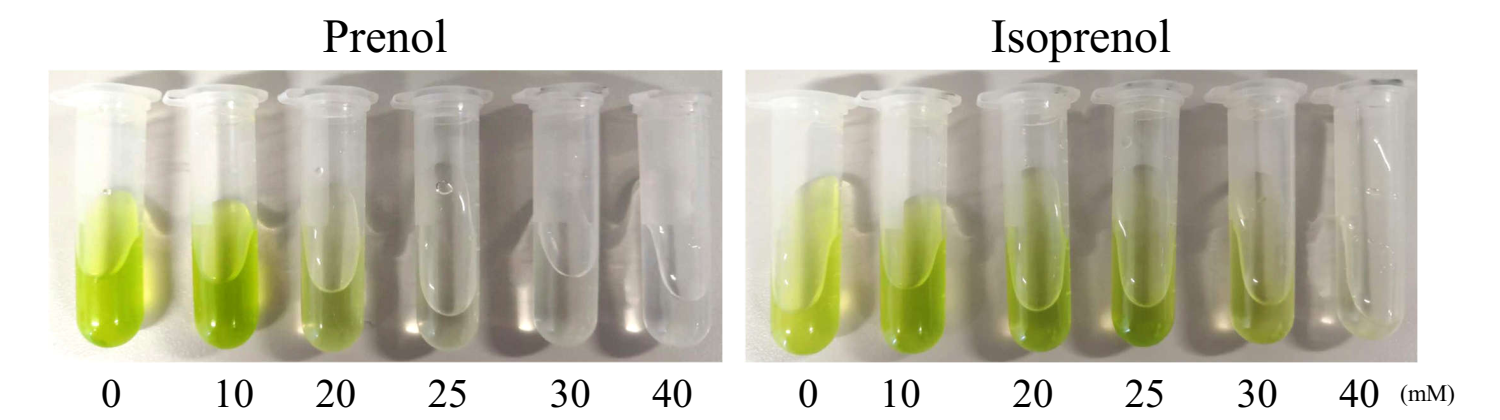

Supplement: Supplementary file 1 [file marinedrugs-20-00577-s001.zip › Figure S1.tif]

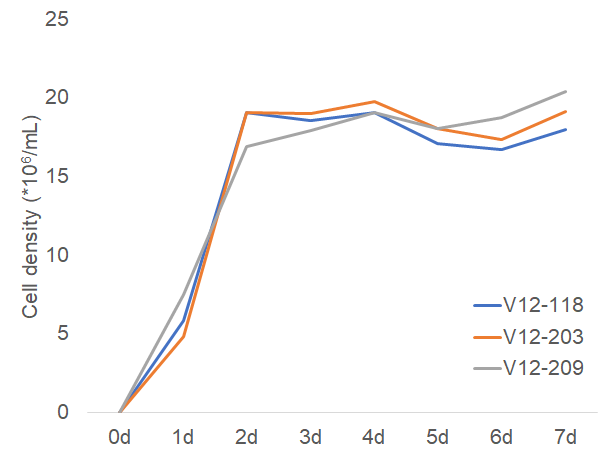

Supplement: Supplementary file 1 [file marinedrugs-20-00577-s001.zip › Figure S2.tif]

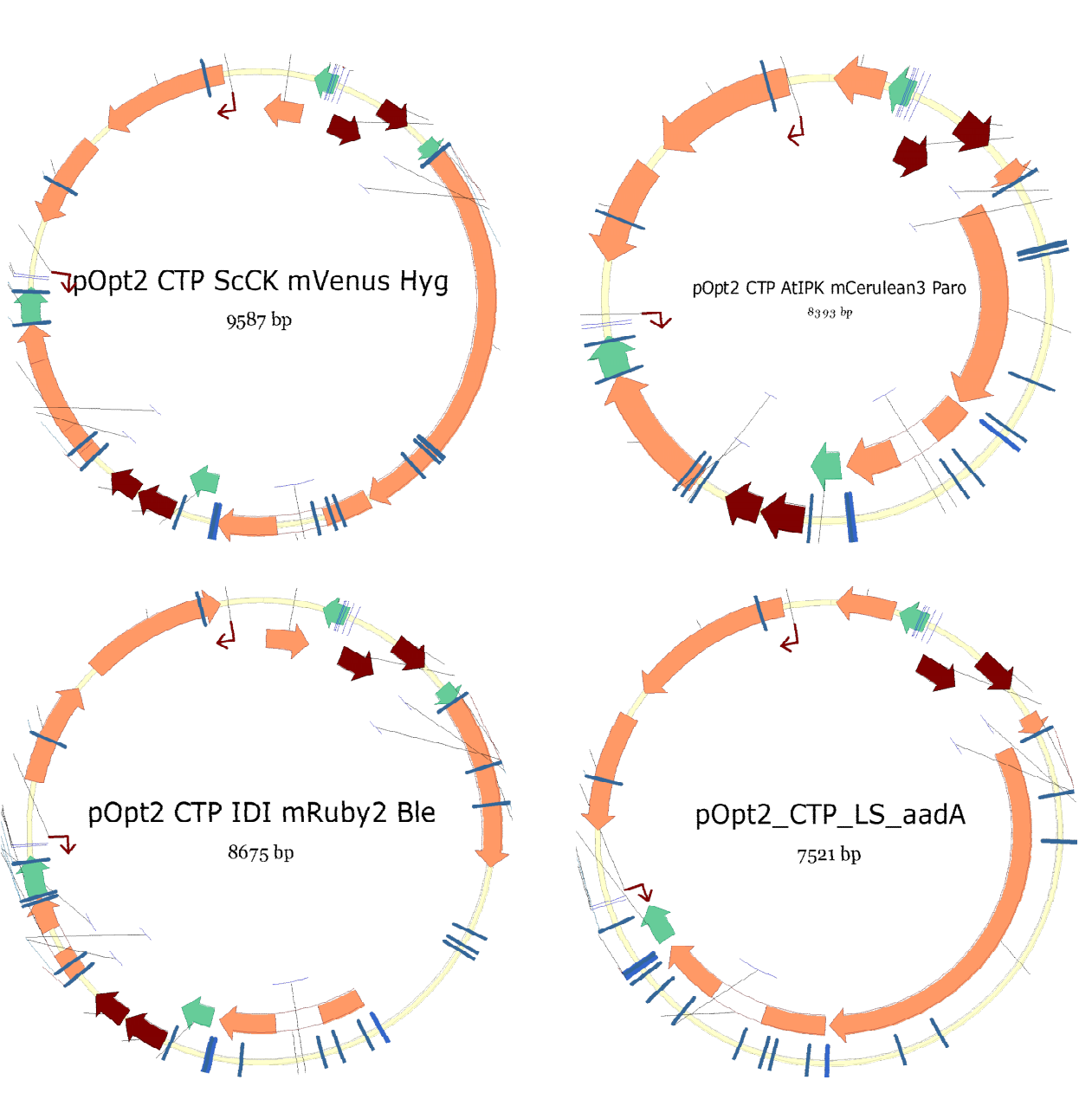

Supplement: Supplementary file 1 [file marinedrugs-20-00577-s001.zip › Figure S3.tif]

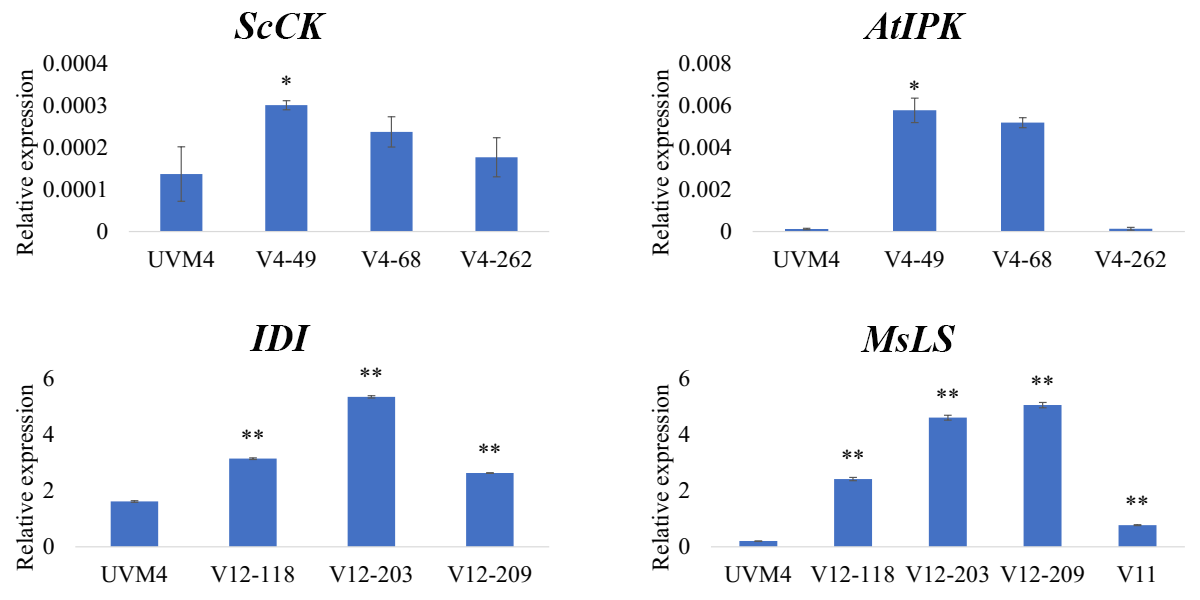

Supplement: Supplementary file 1 [file marinedrugs-20-00577-s001.zip › Figure S4.tif]

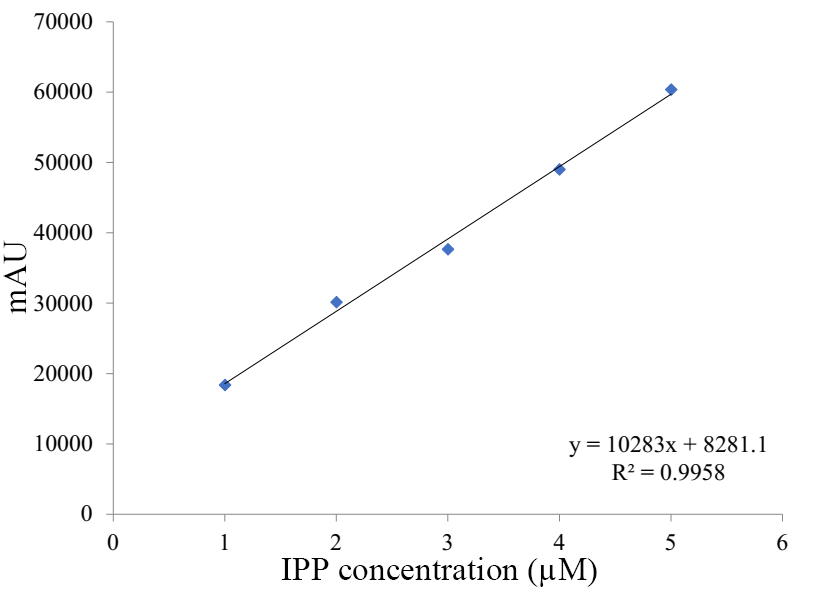

Supplement: Supplementary file 1 [file marinedrugs-20-00577-s001.zip › Figure S5.tif]

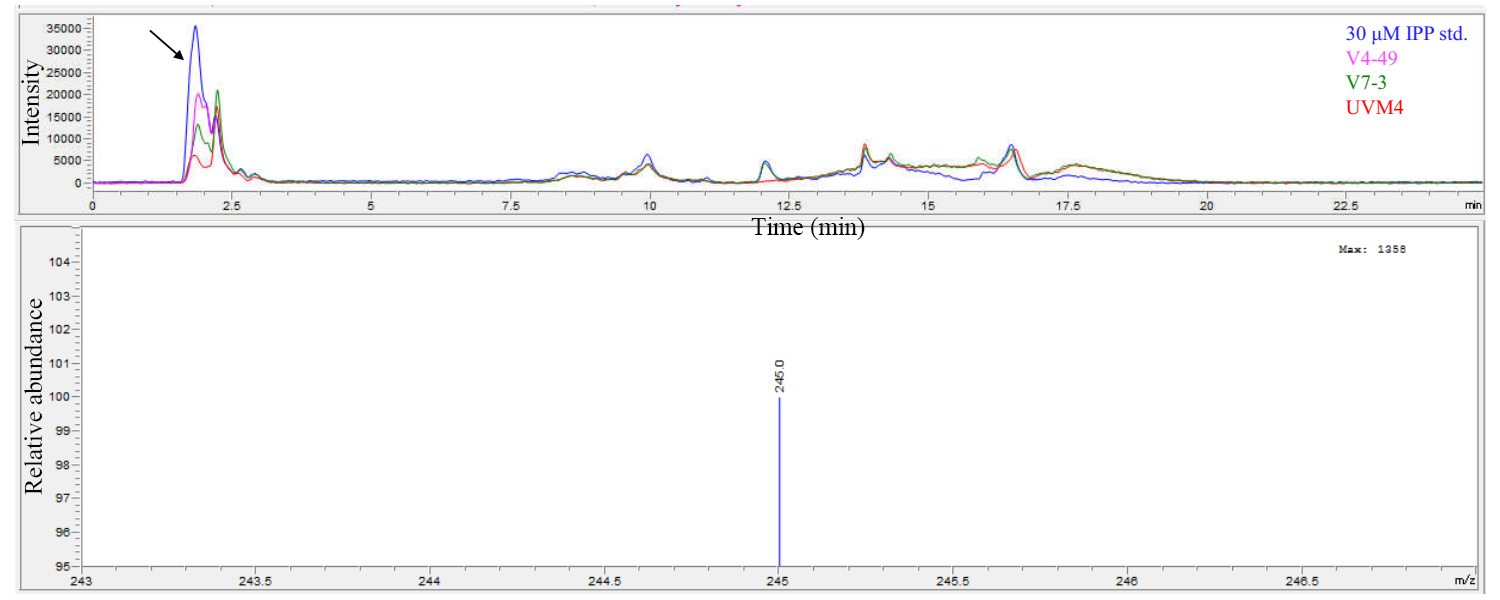

Supplement: Supplementary file 1 [file marinedrugs-20-00577-s001.zip › Figure S6.tif]

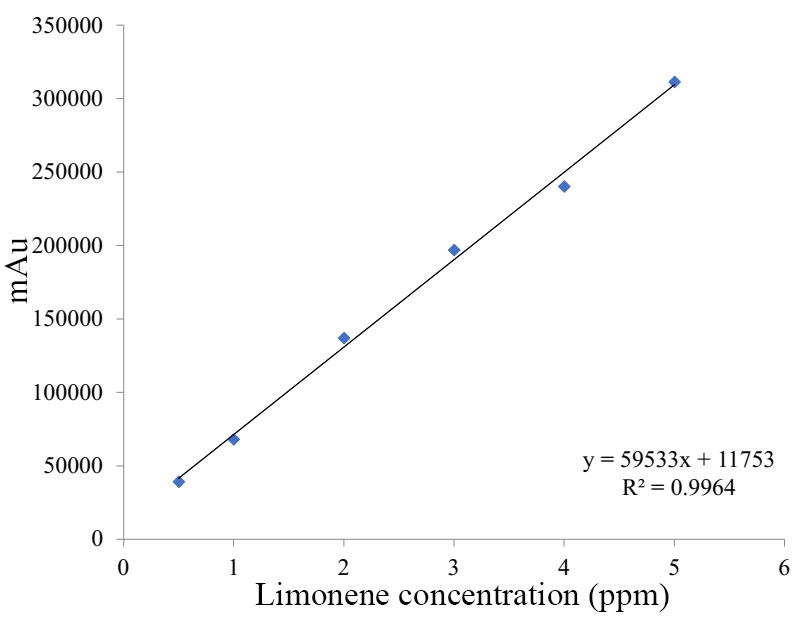

Supplement: Supplementary file 1 [file marinedrugs-20-00577-s001.zip › Figure S7.tif]
